# Effect of γ′ Phase Elements on Oxidation Behavior of Nanocrystalline Coatings at 1050 °C

**DOI:** 10.3390/ma14010202

**Published:** 2021-01-04

**Authors:** Jinlong Wang, Lanlan Yang, Shasha Yang, Yixuan Jia, Minghui Chen, Yanxin Qiao, Pingyi Guo, Shenglong Zhu, Fuhui Wang

**Affiliations:** 1 Shenyang National Laboratory for Materials Science, Northeastern University, Shenyang 110819, China; wangjinlong@mail.neu.edu.cn (J.W.); mhchen@mail.neu.edu.cn (M.C.); fhwang@imr.ac.cn (F.W.); 2 School of Materials Science and Engineering, Jiangsu University of Science and Technology, Zhenjiang 212003, China; yxqiao@just.edu.cn (Y.Q.); pyguo@just.edu.cn (P.G.); 3 Laboratory for Corrosion and Protection, Institute of Metal Research, Chinese Academy of Science, Shenyang 110016, China; ssyang16s@imr.ac.cn (S.Y.); yxjia16s@imr.ac.cn (Y.J.); slzhu@imr.ac.cn (S.Z.)

**Keywords:** superalloys, oxidation, nanocrystalline coatings, magnetron sputtering

## Abstract

To study the effect of γ′ phase elements on the oxidation behavior of nanocrystalline coatings, two comparable nanocrystalline coating systems were established and prepared by magnetron sputtering. The oxidation experiments of the nanocrystalline coatings on the K38G and N5 superalloys were carried at 1050 °C for 100 h, respectively. The chemical composition of the above coatings is the same as the substrate alloy, including the γ′ elements, such as Al, Ta, and Ti. After serving at a high temperature for certain periods, their oxides participated and then affected the oxidation behavior of the coatings. The Al_2_O_3_ scale can be formed on the N5 coating, which cannot be formed on the K38G coating. Tantalum and titanium oxides can be detected on the oxide scale, which ruin its purity and integrity.

## 1. Introduction

Nickel-based superalloys are widely used in aeroengines and gas turbines due to their excellent high-temperature mechanical properties [1,2,3]. They are mainly composed of γ and γ′ phases. The γ phase is a solid solution of Ni, which plays a solid solution strengthening effect [4,5,6]. It shows a face-centered cubic-disordered crystal structure, and can solute Re, W, Mo, and other elements [7,8]. The γ′ phase is the precipitation strengthened phase with a stoichiometric ration of Ni_3_Al. Al, Ti, Ta, Nb, and so on can be dissolved, which are named as γ′ phase elements [8,9,10,11,12]. With the rapid consumption of a large number of alloying element on the high-temperature environment, the original stable γ/γ′ structure will be destroyed [10,11]. Therefore, the long-term service performance of the superalloy in harsh temperatures is often related to its composition components and constitute elements [12]. Thus, in the current superalloy design, the composition of the alloy is often considered and studied to have better high-temperature oxidation resistance.

In addition to the consumption of the γ′ phase suffered by oxidation, this is usually caused by elemental inter-diffusion happening on a coated superalloy. The common usage of high-temperature protective coatings are β-NiAl or MCrAlY coatings, which can be used as bond coating in TBC (Thermal barrier coating) layers or used as separate protective coatings [13,14,15]. After the coating is prepared, the main purpose is to quickly form a protective alumina scale on the alloy surface to achieve protection [16,17]. In this way, it is ensured that the beneficial anti-oxidation elements in the sub-state superalloy will not be consumed excessively, and no structural degradation will occur during the oxidation process. However, due to the great difference between the element content and the alloy composition, the chemical potential gradient leads to the inter-diffusion of elements happening on the interface between the overlay coating and substrate alloy [18,19]. As a result, the γ/γ′ structure in the superalloy is consumed during the interface reaction, and a SRZ (secondary reaction zone) appears beneath the coating. Then, the TCP (topologically close-packed) phase, which has an adverse effect on the mechanical properties of the alloy, will be precipitated in the SRZ [20]. The diffusion reaction of elements in the interface develops very rapidly at a high temperature. It is of great significance to damage the mechanical properties of the matrix or affect the original γ/γ′ structure [21]. Therefore, the research on how to increase the diffusion resistance on the coated alloys attracts worldwide attention.

Nanocrystalline coating has attracted much attention for its excellent high-temperature oxidation resistance [22,23,24,25,26,27,28,29,30,31]. Since Lou invented nanocrystalline coating in 1992, the research of nanocrystalline coating has been developed for nearly 30 years, and more than 30 kinds of alloys have been well-studied [28]. In recent years, the results of diffusion inhibition in a single crystal are more significant [18,22,26,27,28,29,30,31]. The coating is made up of a columnar crystal at nano size, which promotes the rapid diffusion of Al in the coating and the fast formation of the Al_2_O_3_ scale. At the same time, the chemical compositions of the coating are the same with the superalloy, which can avoid a series of problems caused by diffusion [24,28,29,30]. Then, the service life of the coating can be prolonged. The deposited nanocrystalline coating consists only of supersaturated γ phase, in which all elements, including the γ′ phase elements, are dissolved and very unstable [21,22,23,24,25,26]. During service, the γ′ phase elements will be separated from the supersaturated γ phase, and the γ′ phase can be formed. In addition, the γ′ phase elements have high affinity with oxygen. Then, oxide products can be easily formed, which will ruin the integrity and purity of the Al_2_O_3_ scale [21,22,23]. Therefore, its oxidation resistance will be greatly reduced.

In this study, two nanocrystalline coatings were prepared by a PVD (physical vapor deposition) process. The oxidation behavior of nanocrystalline coatings on a typical nickel base alloy K38 and a second single-crystal superalloy N5 were investigated comparably. The effect of γ′ phase elements in the nanocrystalline coating on its oxidation behavior was studied.

## 2. Experiment

The chosen substrate superalloys are the K38G alloy with a high content of Ti and the N5 single crystal superalloy with a high content of Ta, whose chemical compositions are shown in Table 1. The superalloy was designed and produced in the IMR (Institute of Metal Research, Chinese Academy of Sciences, Shenyang, China). Specimens with dimensions of Φ15 mm × 2.0 mm were ground to 2000# SiC papers, and then the samples were ultrasonically degreased in ethanol and acetone for 30 min to obtain a clean surface free of contaminants before coating deposition. The nanocrystalline coatings were prepared by magnetron sputtering for 11 h, with the thickness of the coating approximately 30 μm. The sputtering targets (382 mm × 182 mm × 8 mm) had the same composition as the superalloy substrates. Before plasma sputtering, the vacuum chamber was heated to 200 °C and evacuated to a base pressure below 6 × 10^−3^ Pa. During the deposition, the parameters were set as follows: the working argon pressure was 0.2 Pa, the output current was 3.5 A, and the duty ratio was 80%. The magnetron sputtering parameters are shown in Table 2.

To explore the effect of the γ′ elements on the oxidation behavior of the nanocrystalline coatings, an isothermal oxidation test was adopted at 1050 °C for 100 h. During the whole experiment, the power of the furnace was kept stable, and the temperature inside was fixed at 1050 °C. The temperature in the furnace fluctuated by about ± 1 °C. To ensure the accuracy of the experiment, five parallel samples were selected for each group. The specimens, all placed in crucibles, were taken out of the furnace and cooled to room temperature under a dry atmosphere at various oxidation intervals to record mass gain by an electronic balance (BP211D, Sartorius, Germany) with a sensitivity of 0.01 mg.

The phase constitution and the oxidation products of the nanocrystalline coatings were characterized by XRD (X’Pert PRO, PANalytical Co. Almelo, Holland, OR, USA, Cu Ka radiation at 40 kV). The surface and cross-section microstructures and elemental compositions of the coatings, as well as the thermally-grown oxides layer, were executed by scanning electron microscopy (SEM, Inspect F 50, FEI Co., Hillsboro, OR, USA), coupled with an energy dispersive spectrometer (EDS, X-Max, Oxford instruments Co., Oxford, UK). The fractured cross-sectional morphology was obtained by soaking the deposited coating sample in liquid nitrogen for a certain time, and then the sample was broken with external force to ensure the brittle fracture morphology.

## 3. Results and Discussion

**Figure 1:**
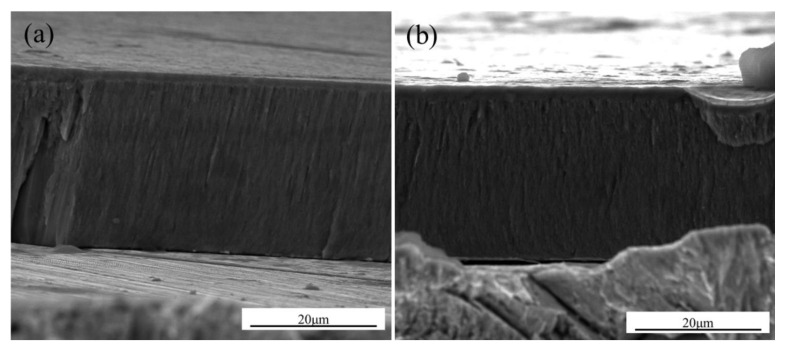
SEM cross-section micrographs of (**a**) K38G and (**b**) N5 nanocrystalline coatings.

The fractured cross-sectional morphologies of the K38G and N5 nanocrystalline coatings are shown in Figure 1. The two coatings exhibited the obvious columnar structure, with a thickness of 29 μm. No obvious difference was detected between the K38G and N5 nanocrystalline coatings. The chemical composition of the two coatings were similar to that of the substrate superalloys. Combined with Table 1, the K38G nanocrystalline coating was rich in Ti, and the N5 coating was rich in Ta.

**Figure 2:**
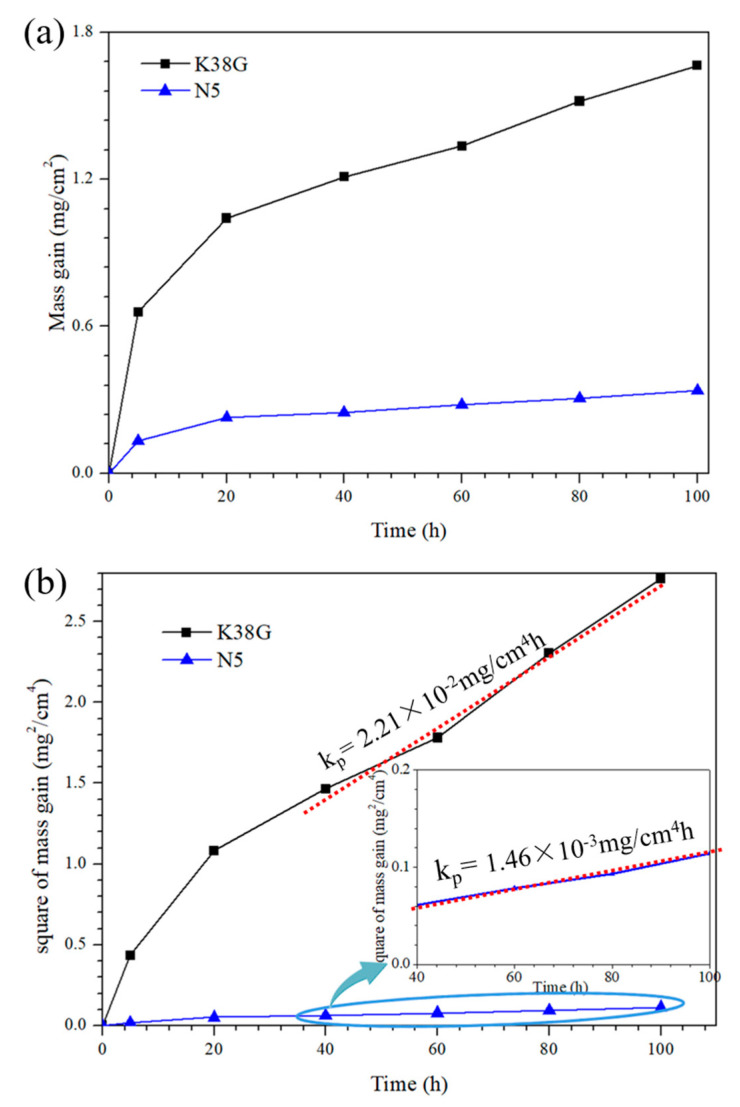
Isothermal oxidation kinetics of the K38G and N5 nanocrystalline coatings at 1050 °C in air; (**a**) y vs. t and (**b**) y^2^ vs. t.

Figure 2 shows the isothermal oxidation kinetics of the K38G and N5 nanocrystalline coatings at 1050 °C. As shown in Figure 2a, both of the coatings show a rapid initial oxidation stage, followed by a slow stabilization stage. After oxidation for 100 h, the mass gain of the K38G nanocrystalline coating was 1.663 mg/cm^2^, while the mass gain of the N5 coating reached 0.338 mg/cm^2^, which was significantly lower than the K38G. All of this indicated that the components and thickness of the oxide scales on the two nanocrystalline coatings should be quite different. To explore the oxidation regulation of the two nanocrystaline coatings, the function relationship between y^2^ and t is plotted in Figure 2b, where y is the mass gain of the coatings and t is the oxidation time. It is clear that both nanocrystalline coatings obey the parabolic law:

(1) $$y^{2}=k_{p}t$$

where k_p_ is the parabolic rate constant. Its value can be directly obtained from Figure 2b. For the N5 nanocrystalline coating, the parabolic constant rate was 4.03 × 10^−7^ mg/cm^4^s, which was similar to that of the NiAl coating, and was covered by α-Al_2_O_3_ [32,33]. These indicated that the α-Al_2_O_3_ was main phase of the oxide scale on the N5 coating. However, for the K38G nanocrystalline coating, the value of the parabolic rate constant was about 6.14 × 10^−6^ mg/cm^4^s, which was one order of magnitude larger than that of the NiAl and N5 coatings. This showed that the main oxide products on the K38G coating were not only α-Al_2_O_3_, but other oxides as well, like TiO_2_ [34,35,36,37].

**Figure 3:**
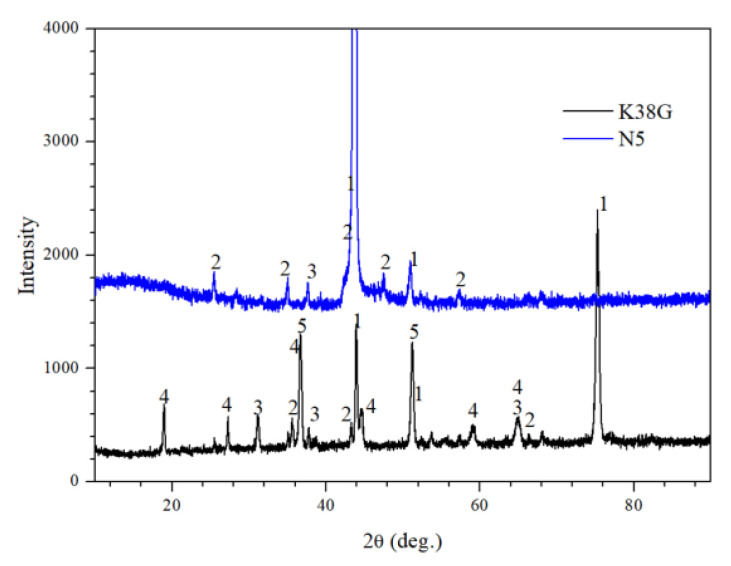
XRD patterns of the K38G and N5 nanocrystalline coatings after oxidation at 1050 °C for 100 h in air (1—γ/γ′, 2—α-Al_2_O_3_, 3—Ta_2_O_5_, 4—TiO_2_, 5—Ni_2_CrO_4_).

To detect the oxide products on the surface of the K38G and N5 nanocrystalline coatings after oxidation, XRD was adopted. As shown in Figure 3, the oxide scale on the N5 coating was mainly composed of α-Al_2_O_3_. Ta_2_O_5_ was also detected on the surface, while for the K38G coating, the oxide products were α-Al_2_O_3_, Ta_2_O_5_, TiO_2_, and Ni_2_CrO_4_.

**Figure 4:**
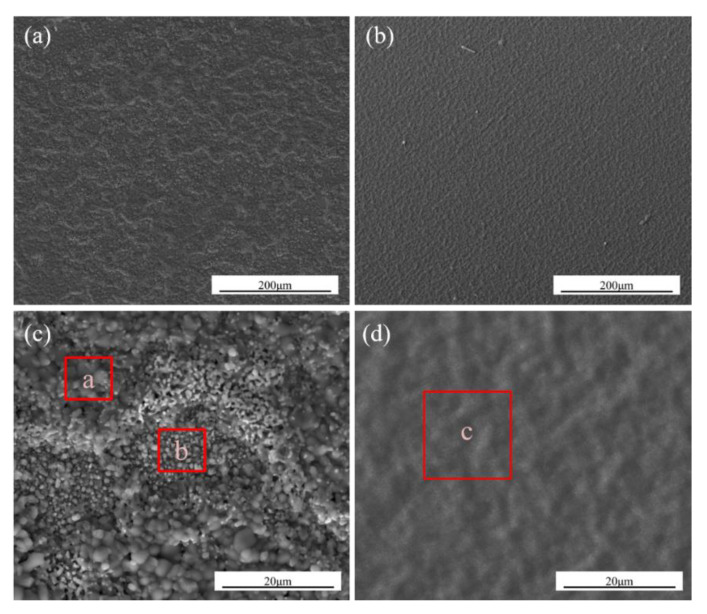
Surface morphologies of the two nanocrystalline coatings after oxidation. (**a**,**c**) K38G alloys; (**b**,**d**) N5 alloys.

Figure 4 shows the surface morphologies of the K38G and N5 nanocrystalline coatings after oxidation at 1050 °C for 100 h. Obviously, both coatings had excellent oxidation resistance, for no cracks or spallation were found, as shown in Figure 4a,b. After oxidation for 100 h, the two coatings showed rough surfaces. It is clear that the oxide scale on the K38G nanocrystalline coating was made up of different kinds of oxides. The chemical compositions of a, b, and c are shown in Table 3. Combined with the XRD patterns in Figure 3, the place of a on the K38G nanocrystalline coating was rich in Ti, and should have been TiO_2_. The point b on the K38 coating and c on the N5 coating were rich in Al, and should have been Al_2_O_3_.

**Figure 5:**
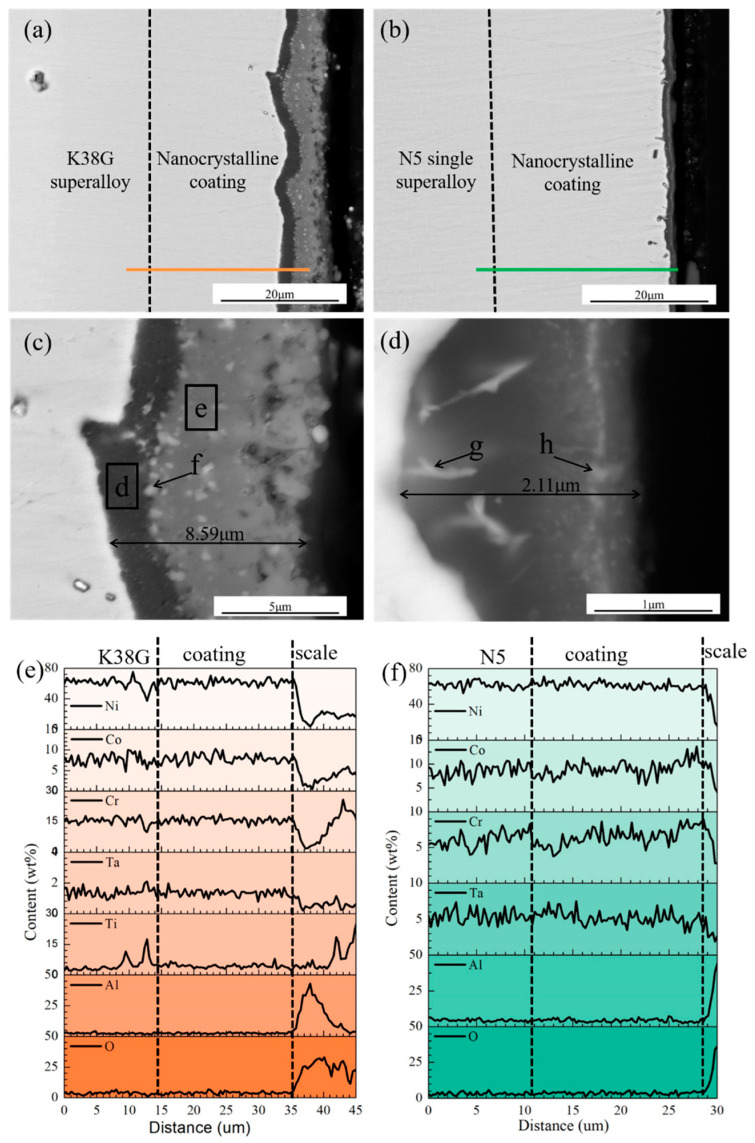
The cross-sectional microstructures of the two nanocrystalline coatings after oxidation. (**a**,**c**) K38G; (**b**,**d**) N5; (**e**) EDS scanning along the red line in (**a**); (**f**) EDS scanning along the red line in (**b**).

The cross-sectional microstructures of the K38G and N5 nanocrystalline coatings after oxidation for 100 h are shown in Figure 5. Oxide scales on the two nanocrystalline coatings were completely different, especially in thickness and composition. The oxide scale on the K38G nanocrystalline coating was about 8.59 μm thick, which was divided into two parts: the dark and grey parts, as shown in Figure 5a. The dark part of the oxide scale was near the K38G coating, and the grey part was near the atmosphere, as shown in Figure 5c. As shown in Table 3, the dark part was rich in Al, and should have been Al_2_O_3_, while the grey part of the oxide scale was rich in Ti, and should have been mainly made up of TiO_2_. Some pores on the oxide scale were detected, denoted by arrows in Figure 5c. Besides these, some white particles were observed. According to the EDS and XRD results, they should have been Ta_2_O_5_. Figure 5e shows the distribution of Ni, Co, Cr, Ta, Ti, Al, and O in the K38G coating and its oxide scale. Ni, Co, Cr, Ta, Ti, Al, and O were evenly distributed in the coating, whose contents were similar to that in the substrate alloy. No element interdiffusion occurred in the K38G substrate beneath the coating.

Combined with the XRD results in Figure 3, the oxide scale on the N5 nanocrystalline coating was mainly composed of α-Al_2_O_3_. The Al_2_O_3_ scale was 2.11 μm thick, as shown in Figure 5e. There was a continuous white stripe composed of many white particles in the middle, as denoted by g and h. According to the XRD results in Figure 3 and chemical compositions in Table 3, these white particles should have been Ta_2_O_5_. The distribution of Ni, Co, Cr, Ta, and Al was even in the coating and the substrate alloy, as shown in Figure 5f. No difference in chemical composition between the N5 coating and alloy was found. To be noted, no interdiffusion zone (IDZ) or secondary reaction zone (SRZ) was detected, which were resulted by element interdiffusion. The difference of the oxidation rate constant indicated the oxidation rate of the coating to a certain extent, that is, the growth rate of the oxide scale. The oxidation rate constant of the N5 nanocrystalline coating was obviously smaller than that of K38G, which indicated that the oxidation rate of the N5 coating was slower, and the growth rate of the oxide scale on the coated specimen was slower. Therefore, the oxide scale on the surface of the N5 nanocrystalline coating was thinner than that of the K38G coating after oxidation for 100 h. In addition, the different oxidation rate constants indicated that the oxidation products on the coating surface will be different. For example, the oxidation rate constant of the N5 coating was closer to that of the NiAl coating, and the surface of the coating was covered by α-Al_2_O_3_ at the temperature in this work. It can be indicated that Al in the N5 coating was mainly involved in oxidation to form an alumina scale. The oxidation rate of the K38G coating was much higher than that of the N5 coating, which indicated that more active elements in the K38G coating participated in the oxidation reaction. These were consistent with the XRD results.

**Figure 6:**
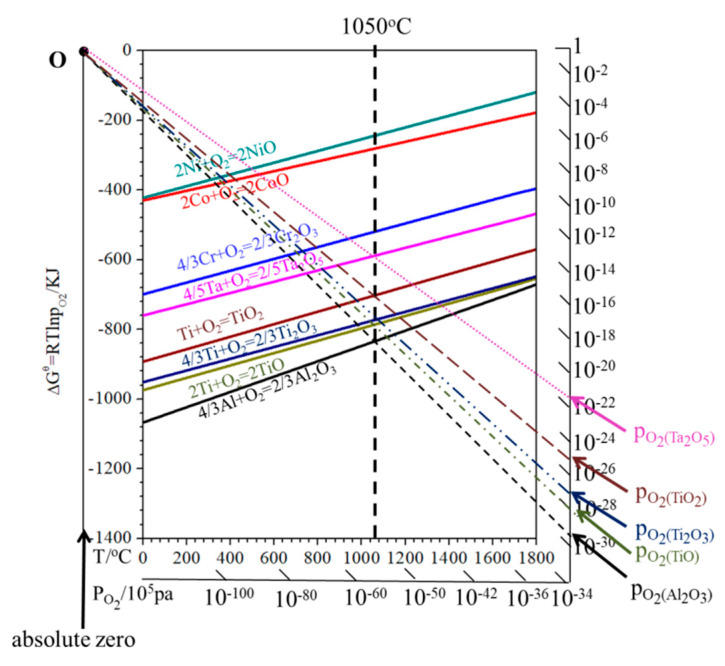
Ellingham diagram of Ni, Co, Cr, Ta, Ti, and Al (per mole O_2_).

The oxide scale on the K38G nanocrystalline coating was composed of α-Al_2_O_3_, Ta_2_O_5_, TiO_2_, and Ni_2_CrO_4_; α-Al_2_O_3_ and Ta_2_O_5_ were also detected on the N5 nanocrystalline coating surface. All of these seriously deviated from the original design intention of the nanocrystalline coating, which was expected to form an Al_2_O_3_ scale. To solve the above problems, the Ellingham diagram [18] of Ni, Co, Cr, Ta, Ti, and Al is shown in Figure 6. It was inferred that ∆G of the Al_2_O_3_ was the lowest, followed by the oxides of Ti and Ta. For the N5 nanocrystalline coating, Al and Ta were the main γ′ elements. Combined with the Ellingham diagram and the contents of Al, the Al_2_O_3_ scale will be preferentially formed on the coating surface, which was already confirmed. At the same time, the columnar crystal at nano size in the coating was conducive to the rapid diffusion of Al, and promoted the formation of the protective Al_2_O_3_ scale. With the consumption of Al, its content decreased, while the content of Ta increased, and would be oxidized. Due to the fact that the P_O2_ of Ta_2_O_5_ is higher than that of Al_2_O_3_, the tantalum will move to the surface of the Al_2_O_3_ scale. However, due to the large size of the Ta_2_O_5_, it cannot move the surface [26,27,28,29,30,36,37,38,39,40].

In the initial stage, the oxidation product formed on the K38 nanocrystalline coating was Al_2_O_3_. Although the K38 alloy is designed as a Cr-forming superalloy at high-temperature oxidation, the content of Cr (16 wt%) was much higher than that of Al (4 wt%) in the substrate. However, due to the special nanocrystalline structure of the coating, the selective oxidation ability of the Al element improved, and the protective oxidation products were formed at the initial stage; this has been confirmed by our earlier study [40]. However, due to the limited content of Al, the Al depletion area will be formed after a certain period of time beneath the oxide scale, and the other elements will be oxidized. The calculated results of the XRD and Ellingham diagram also confirmed that, except for Al_2_O_3_, Ti, Ta, Cr, and Ni, oxides would be oxidized and participate in the oxide scale. As the temperature at 1050 °C was too harsh for these elements to form an intact and protective scale, the oxidation products formed on the coated specimens had many defects, such as the existence of some cavities or gaps observed in Figure 4 and Figure 5. The oxidation process will gradually become unstable with the extension of time. Some NiCr_2_O_4_ spinel were formed by the reaction of Cr_2_O_3_ and NiO, which become the dominant oxidation products, and then, a variety of oxide products will be formed on the surface. The main γ’ elements were Al, Ti, and Ta, and their contents were too low to form a protective oxide scale in the K38 nanocrystalline coating. Due to the high content of Ti, the oxide layer dominated by titanium will be formed, as shown in Figure 5c. With the consumption of Ti and other elements in the coating, Al and Ta contents will increase. Next, the effect of Ta and Al on the oxidation behavior was the same as that of the N5. In the later stage, as the P_o2_ at the interface of the sub-state and oxide scale decreased to a certain extent, the alumina would be precipitated. Then, the inner Al_2_O_3_ scale is formed, and Ta_2_O_5_ can be detected in the oxide scale.

## 4. Conclusions

From the above study, the following conclusions can be drawn:

(1) The high-temperature oxidation resistance of the nanocrystalline coating on the single crystal superalloy of N5 is better. When the coating is serviced at 1050 °C, it will be covered by Al_2_O_3_.
(2) Ta contained γ’ phase in the N5 nanocrystalline coating, which ruined its oxide’s purity and integrity after oxidation.
(3) The oxide scale of the K38G nanocrystalline coating is divided into two parts: the inner one is made up of Al_2_O_3_, and the outer one is mainly composed of NiCr_2_O4_2_. In addition, TiO_2_ and Ta_2_O_5_ can be detected.
(4) Excessive γ′ elements will affect the purity of the oxide scale on nanocrystalline coatings, and then affect the oxidation kinetics and microstructure results.

## Figures and Tables

**Table 1:** Chemical compositions of the chosen Ni-based superalloys (wt%).

Elements	Al	Ti	Ta	Co	Cr	Mo	W	Re	Ni
K38G	3.0	3.5	1.5	9	16	1.5	2.5	0	Bal.
N5	6.2	0	6.5	7.5	7	1.5	5	3	Bal.

**Table 2:** Sputtering parameters for nanocrystalline deposition.

Parameters	Value
Argon pressure	0.2 Pa
Current	3.5 A
Temperature	200 °C

**Table 3:** Chemical composition a, b, c, d, e, f, g, and h in Figure 4 and Figure 5 by EDS (wt%).

Elements	Ni	O	Al	Ta	Ti	Cr	Co
a	24.8	34.6	6.8	1.4	15.0	15.0	2.4
b	25.7	33.4	20.3	2.6	2.5	10.7	4.8
c	11.9	40.3	39.9	1.8	0	3.6	2.5
d	6.8	45.1	42.2	0.9	2.0	1.8	1.2
e	14.4	42.3	3.6	0.7	25.0	10.8	3.2
f	14.1	46.0	26.0	4.8	5.3	2.6	1.2
g	3.2	41.3	35.8	18.1	0	1.1	0.5
h	4.5	40.7	33.2	20.3	0	0.7	0.6

## Data Availability

Data sharing not applicable.
